# Are individuals who are positive about artificial intelligence also more unsure?

**DOI:** 10.1016/j.patter.2025.101374

**Published:** 2025-10-10

**Authors:** Moinak Bhaduri

**Affiliations:** 1 Department of Mathematical Sciences, Bentley University, Waltham, MA, USA

## Abstract

As artificial intelligence matures, the impact it might have on how society functions is being actively pondered. In this opinion, through uniform-binomial mixtures, the author sheds quantitative light on the matter, showing topics that unite and divide the population on an unobserved, latent level.

## Main text

Wide applicability, though coveted, can be a double-edged sword. The ways in which a tool becomes applicable in a variety of contexts might be the very channels through which it could attract resentment should one of these contexts earn a bad name for itself. The applications of data science (DS) in artificial intelligence (AI) are undeniable. If consumers’ relationship with AI becomes frosty, through the self-harm-inflicting edge of that sword, some of that coldness may seep in toward DS, purely due to *association*.

Where I work, we have been (jointly with Gallup) designing surveys to document people’s opinions on a vast array of business and social topics over the last three years (https://www.bentley.edu/gallup). People’s thoughts on AI’s applicability in interesting situations are recorded on a Likert scale—please see Table 1 for eight of these topics—along with demographic details such as their age, gender, racial background, and political leanings. In the 2024 round, there were 5,835 respondents. Previously,^1^ I have investigated non-AI topics through tools such as Markov random fields to point out how strengths of automatic connections between two tendencies (like offering fair wages to employees and staying mindful of climate impacts) vary substantially across political and age divides, how these differences are mainly of magnitude instead of kind, and how, despite these differences, commonalities persist. With Bayesian posteriors,^2^ we noted which demographic (age-political combination) remained generally upbeat about modern businesses practices in integrated social systems. In addition to these academic works, we have been disseminating these insights^3^^,^^4^ through contemporary media using methods such as writing peer-reviewed explanatory articles, creating interactive dashboards, and appearing on podcasts (https://statsandstories.net/economics1/how-businesses-function). Tacit in the above body of work was the assumption that the recorded answers were *exact*. When a respondent reported feeling “extremely negatively” toward a topic, they *truly* felt extremely negatively. The chief merit of the current piece is in augmenting these earlier works through querying that assumption: in making provisions for Likert scale respondents to be fundamentally indecisive, in pointing out the way that indecisiveness manifests, and in showcasing to interested practitioners the workings of an easy-to-implement statistical model that allows answers to emerge entangled with objective feelings and fuzzy uncertainties.

**Table 1:** Opinion and non-opinion questions and responses on the Bentley-Gallup Force for Good survey, wave 3, 2024

Question	Answer	CUB $\left(\hat{\pi },\hat{\xi }\right)$; GOF *F*
**Opinion**		

Q54: How knowledgeable are you about artificial intelligence?	Not at all (1), Not very (2), Somewhat (3), Extremely (4)	(0.9990, 0.4499); 0.8367
Q44: What type of effect will artificial intelligence have on the total number of jobs in the US over the next 10 years?	Increase the number of jobs (1), Have no impact (2), Reduce the number of jobs (3)	not ideal for 3-way responses
Q55A: How concerned are you about AI being used to recommend financial advice?	Extremely concerned (1), Somewhat concerned (2), Not very concerned (3), Not concerned at all (4)	(0.9792,0.6604); 0.9841
Q55B: How concerned are you about AI being used to recommend medical advice?	Extremely concerned (1), Somewhat concerned (2), Not very concerned (3), Not concerned at all (4)	(0.9514,0.7380); 0.9717
Q55F: How concerned are you about AI being used to drive a car?	Extremely concerned (1), Somewhat concerned (2), Not very concerned (3), Not concerned at all (4)	(0.8701, 0.8199); 0.9563
Q55H: How concerned are you about AI being used to recommend which employees to hire?	Extremely concerned (1), Somewhat concerned (2), Not very concerned (3), Not concerned at all (4)	(0.9455, 0.7912); 0.9890
Q55I: How concerned are you about AI being used to assist students with homework or studies?	Extremely concerned (1), Somewhat concerned (2), Not very concerned (3), Not concerned at all (4)	(0.8781, 0.6604); 0.9550
Q55K: How concerned are you about AI being used to create original artistic content?	Extremely concerned (1), Somewhat concerned (2), Not very concerned (3), Not concerned at all (4)	(0.6799, 0.7469); 0.9386

**Demographic/political**		

How would you describe your political views?	Very conservative, Conservative, Moderate, Liberal, Very liberal	–
Gender	Male, Female, Non-binary	–
Age	Age in years: Young (<34), Middle-aged (35–57), More mature (>58)	–
What is the highest level of school you have completed or the highest degree you have received?	Low (less than high school degree or diploma), Moderate (some college courses, associate, bachelor’s), High (master’s, professional, doctorate)	–
Race	White, Other, Black, Asian, Hispanic	–

Estimated parameters from CUB models are shown in the last column, along with goodness-of-fit (GOF) statistics (*F*). The model and these parameters and statistics are introduced in Bhaduri,^1^ later in the article.

We told respondents the working definition: by AI applications, we mean instances when a computer does or enhances a task that is normally performed by a person. When a participant voices an opinion about an AI topic on an ordered 1-to-4 scale, that response is typically the upshot of a *feeling* attitude toward the topic and an inherent *uncertainty* or unsureness around that decision. Statistically, the recorded response is a combination of a random variable that explains the feeling part and another that explains the uncertainty part. In this opinion, I pinpoint which variable was in control, which one had the bigger share. Put differently, given the response distribution to a raised AI issue, I unearth the default uncertainty (and, hence, certainty) toward the topic that is most likely to have generated that response signature.

Formally, combined uniform-binomial (CUB) models^5^ fit rating data through “mixing” a shifted-binomial V(m,ξ) distribution, with success chance 1−ξ, modeling the “feeling” piece and a discrete uniform U(m) distribution modeling the “uncertainty” piece. Here, m represents the number of ordered responses: 4, in this study. A CUB random variable R, therefore, has a probability mass function

$$P\left(R=r|\vec{\theta }\right)=\pi \left(\begin{array}{c} m-1\\ r-1 \end{array}\right)\xi^{m-r}{\left(1-\xi \right)}^{r-1}+\left(1-\pi \right)\frac{1}{m}\text{,}$$

with $r=1,2,\ldots ,m\;\text{and}\;\vec{\theta }=\left(\pi ,\xi \right),0<\pi \leq 1,0\leq \xi \leq 1\text{.}$

Note that a high share on feeling (i.e., a high π) or, equivalently, a low reliance on unsureness (i.e., a low (1−π)) need not mean that we expect more positive ratings (like 3s or 4s). It means only that we are leaning toward precision—however people feel about that topic, positively or negatively, they are very sure of that outlook. Put differently, as π→0, *R* tilts toward a discrete *uniform* random variable, reflecting people’s total unsureness about how they feel about that topic, much like randomly choosing a rating without thinking at all. Note that π is allowed to touch 1 (i.e., when people are not uncertain at all) but not 0, since that will annihilate the first component and we will not have a truly mixed distribution. I point readers to Arboretti et al.^6^ to explore how these CUB models are used to estimate customers’ unsureness in food packaging surveys. If π is fixed, bigger values of ξ will give higher probabilities to lower values of *R*: in this way, ξ reflects “feelings.” In the context of question 55, for instance, this reflects people’s negative feelings toward the topic. The parameters (π,ξ) are estimated through maximizing the log likelihood

$$l\left(\theta \right)=\sum_{i=1}^{n}logP\left(R=r_{i}|\theta \right)=\sum_{i=1}^{n}\log\left\{\pi \left(\begin{array}{c} m-1\\ r_{i}-1 \end{array}\right)\xi^{m-r_{i}}{\left(1-\xi \right)}^{r_{i}-1}+\left(1-\pi \right)\frac{1}{m}\right\}$$

numerically. I record these estimates for all the AI topics in the last column of Table 1. Next, goodness-of-fit-type investigations are natural, so I query how close the agreements are between the observed (relative) frequencies of the response types with theoretical probabilities generated from CUB models made with these estimated parameters. Chi-squared tests on contingency tables that measure the gap between the observed number of 1s, for instance, and the expected number under the model are known to be unreliable^5^ for ordinal data. Therefore, I also record in Table 1 the *F* index, the complement of a normed dissimilarity that measures the proportion of respondents rightly predicted by the estimated CUB model

$$F=1-\frac{1}{2}\sum_{r=1}^{m}\left|f_{r}-p_{r}\left(\hat{\pi },\hat{\xi }\right)\right|,0\leq F\leq 1\text{,}$$

where $f_{r}$ are the observed relative frequencies and $p_{r}\left(\hat{\pi ,}\hat{\xi }\right)$ are the probabilities estimated by the CUB models for the rth response category. Values close to 1 are desirable, as they indicate close similarities between the observed and expected frequencies of each response type.

The estimated parameters—documented in the final column of Table 1—bring out interesting insights. These results are for the general population, i.e., without regard to an individual’s demographic details or political leanings. Recall that large estimated values of π and low values of ξ for a topic mean people are very sure (using π) that they are giving high Likert scores (using ξ) to that issue. The application is surely (using π) very favorable (using ξ), in a way, for the 55-set. We observe a definite grouping of these issues. With regard to AI’s application in the areas that are generally perceived to be life-altering—such as offering financial or medical advice or hiring employees—respondents are more sure of their answers (high π). Initially, the proliferation of DS and AI must have been a dizzyingly happy affair. As days have gone by and AI has tried to venture into other areas such as helping students with their homework or creating artistic content, much of that excitement has worn off, and the lower πs could point to an erosion of the public level of trust, showing a deep-seated crisis of confidence in these more emerging themes. This public skepticism could represent the final throttling of a promise that seemed potent at its inception.^7^^,^^8^

**Figure 1:**
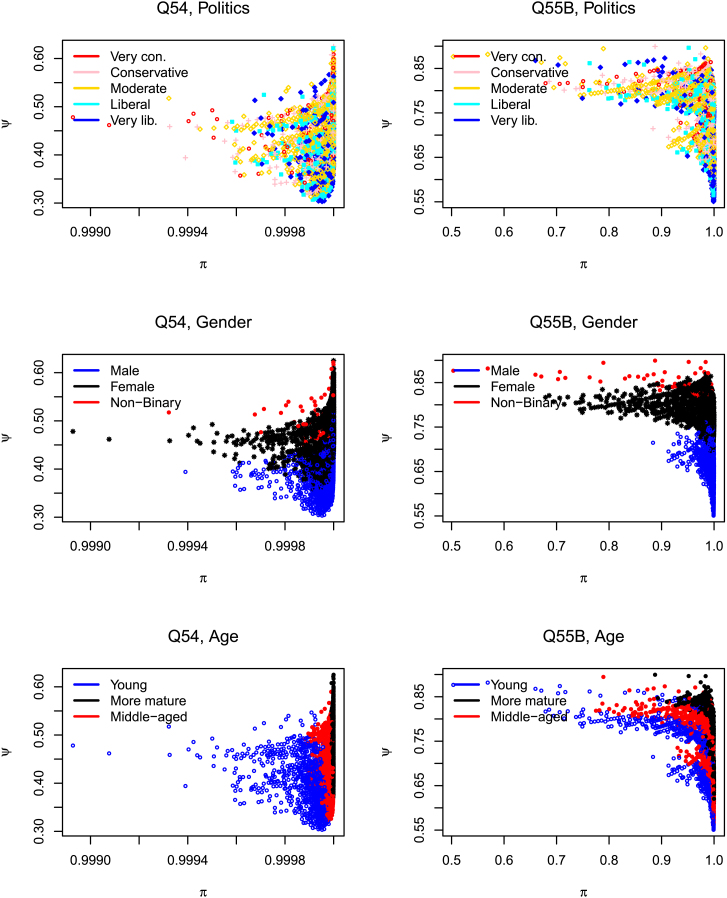
Scatterplots documenting the correlational “field” generated by “uncertainties” (horizontal axes) and “feelings” (vertical axes) Note instances where the forces co-vary (Q54) and where they anti-vary (Q55B). Data behind each panel are available on the author’s GitHub and figshare^10^^,^^11^ accounts along with individual panels that will enable readers to investigate a question-detail combination in isolation or in comparison with other question-detail combinations.

**Figure 2:**
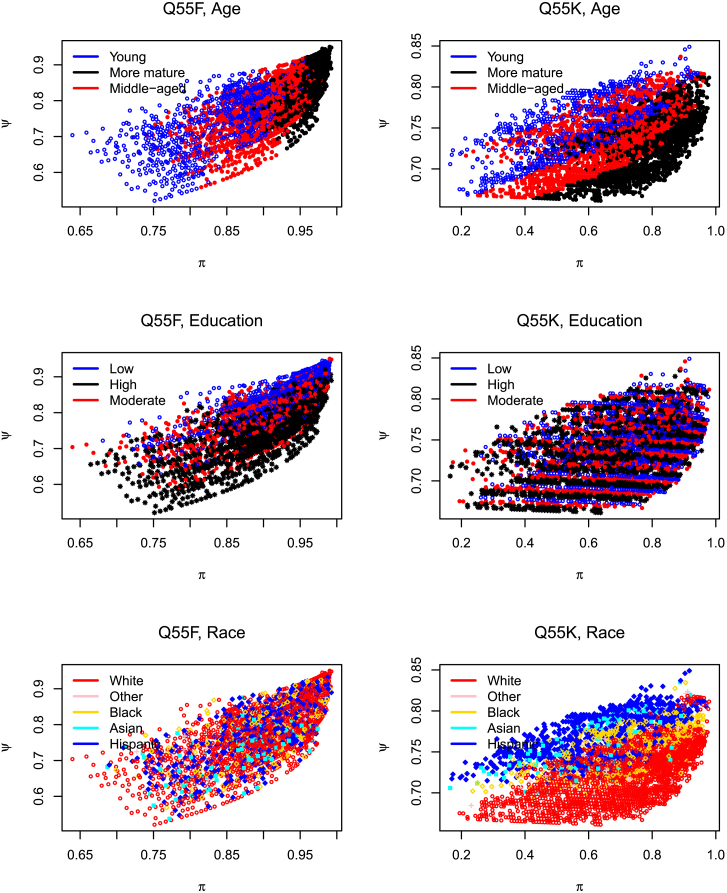
Profile volatilities fluctuate as issues alter Separation of details often translates to separation of unsureness-feeling profiles (for instance, on Q55F). Interesting observations are recorded and detailed in the main text. For instance, Q54 (Figure 1) generally has high estimates of π, while question 55K has low estimates. Data behind each panel are available on the author’s GitHub and figshare^10^^,^^11^ accounts along with individual panels that will enable readers to investigate a question-detail combination in isolation or in comparison with other question-detail combinations.

I next turn to possible dependencies between uncertainty and feeling by extracting estimated parameters at the individual level, i.e., the (π,ξ) parameters for *each* responding person. The pairs are shown in series of scatterplots in Figures 1 and 2. I have regressed the parameters on the five demographic and political details (shown in Table 1) and recorded these insights, through colors, on the resulting plots. Each dot represents an individual’s certainty (on the horizontal axis) and feeling (on the vertical axis) toward a topic, and relevant details about the individual may be recovered by slicing out the appropriate panel. For example, if we ignore the color on the leftmost dot on Figure 1, question 54, we know of a person who is less sure that they are knowledgeable about AI (in comparative terms, i.e., relative to the other individuals on this theme). However, if we vary the colors (i.e., move across Figures 1A–1E) we construct a profile of this individual: we know this is a (politically) very conservative young woman who has low(er) formal education and who is white. Note:

(1) The overall form of the scatter pattern alters as we change topics (regardless of demographic or political details): on most of the 55-set, increasing sureness of responses is generally accompanied by increasing negative feelings toward the topic, while on 55B, sureness is accompanied by positive feelings. On some topics, therefore, the “unsureness” and “feeling” forces co-vary; on others, such as offering medical advice (55B), they anti-vary.
(2) Separation of details often accompanies separation of uncertainty-feeling profiles. (This bolsters some of our earlier works, where we noted substantial separation of the *observed* responses, thereby supplying a firmer foundation.) To the best of my knowledge, similar tendencies have not been explored in social surveys designed in like settings. Regardless of whether there is (like politics, age, or education) or is not (like gender or race) a gradation of details, similar individuals tend to congregate similarly on the uncertainty-feeling two-dimensional (2D) plane. This clustering effect is more pronounced on topic I or F but less apparent on the others. The stronger (i.e., the neater) the clustering, the easier it is to match a probable effect with a profile.
(3) In each panel, certain aspects of these scatterings are expected (such as on question 54, where older respondents are sure they are less knowledgeable about AI and people with “higher” education are sure that they are more knowledgeable; there is a neat transition across age or education profiles), while some are not (that women almost surely feel they are less knowledgeable about AI than their male counterparts; that there are distinct clusters of the same color, such as the red, on 55B, for instance).
(4) To those inclined that way, these patterns may strike a chord around AI’s philosophical basis. For instance, analytical idealists such as Bernardo Kastrup^9^ lay conscious beings in an analogous 2D map, where “intelligence” is plotted on the horizontal axis and “consciousness” on the vertical. To those who ponder whether AI could/should become conscious, these thinkers point out an inverted parabola, suggesting that to make AI “conscious,” we may have to sacrifice some of its “intelligence.”

The goodness-of-fit statistics shown in the last column of Table 1 are close to 1, suggesting the closeness of the observed and expected frequencies, essentially confirming the validity of these patterns and trends. There may be endless mathematical proposals from this point on, offering modified models, along with excursions into subcombinations of demographic and political categories. Such analyses are always promising, and in this opinion, I supply some preliminary insights hoping to intrigue rather than exhaust the reader.

Understanding fluctuations in *objective* measurements—such as an object’s length or its weight—is simplified by objectivity: as the measuring instrument gets more and more accurate, variations diminish. Such variations are at the observed level. While dealing with opinions, however, we need to contend with variations at the unobserved (i.e., latent or inherent) level, too. We are unable to shut off portions of our subjective experience or fuzzy expectations and focus on the objective elements of an AI issue. We react to these topics in the fullness of our imperfections. π’s job is just that: to measure the degree of imprecision, the amount of imperfection. In time, π may tend to 1 as we become surer and surer of AI’s impact (similar to our sureness about how exercising is beneficial to our health). Until then, these uniform-binomial mixtures, by expressing a recorded attitude as a combination of an inbuilt uncertainty and feeling toward an AI issue, may supply a clarification of opinion differences, a momentary stay against confusion.

### Statement on survey design, data and code availability, and software codes

The 2024 Bentley-Gallup Business in Society study is based on a Gallup Panel web survey completed by 5,835 adults, aged 18 and older, in the US that was conducted April 29, 2024, through May 6, 2024. The Gallup Panel is a probability-based longitudinal panel of US adults whom Gallup selects using random digit dialing phone interviews that cover landlines and cell phones. Gallup also uses address-based sampling methods to recruit panel members. The Gallup Panel is not an opt-in panel. The sample for this study was weighted to be demographically representative of the US adult population, using the most recent Current Population Survey figures. For results based on this sample, the margin of sampling error at the 95% confidence level is ±2.1 percentage points for response percentages around 50% and is ±1.3 percentage points for response percentages around 10% or 90%, design effect included. Margins of error are larger for subsamples. More at this link (with some simple descriptive statistics as well): https://www.bentley.edu/gallup.

Readers interested in accessing the full data (and variable descriptions) are encouraged to contact Bentley University’s Academic Technology Center at atc@bentley.edu. Illustrative R codes behind the statistical analyses showcased here, along with the *x*-*y* coordinates of the data dots, are available at the author’s GitHub and figshare^10^^,^^11^ accounts: https://github.com/moinakbhaduri/BentleyGallupAI.CUB.Analysis and https://doi.org/10.6084/m9.figshare.29651945.

## Acknowledgments

M.B. is funded by the Research Enhancement Grant (2024–2027 cycle) awarded by the American Mathematical Society and the Simons Foundation. Bentley University’s summer support and funds from its research council, faculty affairs committee, and provost’s office are also gratefully acknowledged.

## Declaration of interests

The author declares no competing interests. https://www.bentley.edu/gallup lays out the details of the Bentley-Gallup collaboration.

## Declaration of generative AI and AI-assisted technologies in the writing process

The author certifies that no artificial intelligence was used in this project, neither while writing the opinion nor while conducting the analyses.
